# A multidisciplinary study on *Clinostomum* infections in Nile tilapia: micro-morphology, oxidative stress, immunology, and histopathology

**DOI:** 10.1186/s12917-024-03901-7

**Published:** 2024-02-20

**Authors:** Olfat A. Mahdy, Sahar Z. Abdel-Maogood, Mohamed Abdelsalam, Mai A. Salem

**Affiliations:** 1 Department of Parasitology, Faculty of Veterinary Medicine, Cairo University, PO 11221, Giza, Egypt; 2 Department of Aquatic Animal Medicine and Management, Faculty of Veterinary Medicine, Cairo University, PO 11221, Giza, Egypt

**Keywords:** Yellow grub disease, *Clinostomum* species, SEM, *Oreochromis niloticus*, Oxidative stress factors, Immunological cytokines, Histopathological alterations

## Abstract

Yellow grub disease, caused by *Clinostomum* metacercaria, is an endemic zoonotic infection in freshwater fish, responsible for Halzoun syndrome transmitted through the consumption of raw infected fish. This study aimed to conduct a multidisciplinary investigation integrating detailed morphology, oxidative stress, immunology, and histopathology alteration to advance our understanding of Clinostomum infection. In this annual study, 400 Nile tilapia (*Oreochromis niloticus*) were collected from the Nile River at Al Bahr Al Aazam, Giza Governorate to assess *Clinostomum* infection prevalence. Of the examined fish, 160 individuals (40.0%) harboured larval *Clinostomum* infections. *Clinostomum* metacercariae were observed in various anatomical locations, with 135 fish (33.8%) in buccal cavities, 21 fish (5.25%) in gill chambers, and 4 fish (1.0%) on the skin. Infection intensity ranged from 2 to 12 cysts per fish, averaging 5 cysts, notably with skin infections characterized by a single cyst in each fish. Macroscopic encysted metacercariae were collected from buccal cavities, gills, and skin. Micro-morphology revealed distinct features in *C. complanatum*, including an elliptical oral sucker with collar-like rings and large sensory papilla-like structures, contrasting with the absence of these features in *C. phalacrocoracis*. Oxidative stress, assessed through malondialdehyde (MDA) and nitric oxide levels, revealed an elevation in MDA to 35.13 ± 6 nmol/g and nitric oxide to 25.80 ± 3.12 µmol/g in infected fish. In infected fish, MHC-I gene expression increased approximately 13-fold, MHC-II peaked at 19-fold, and IL-1β significantly upregulated by 17-fold, compared to control levels. Histopathology detailed associated lesions, such as cyst encapsulation and eosinophilic infiltration. Clinstomiasis and its impacts on fish hosts offer crucial insights to control this emerging fish-borne zoonotic disease, threatening wildlife, aquaculture, and human health.

## Introduction

Yellow grub diseases, caused by *Clinostomum* metacercariae in freshwater fishes is a matter of pose a significant global threat, particularly for human health. *Clinostomum* EMCs are digenetic trematodes with genetic heterogeneity and complex life cycle, including two hosts: snails and fish. Their larval stages significantly affect the aquaculture industry, especially due to infection with larval stages in the second intermediate host (fishes) [1, 2]. *Oreochromis niloticus* (Nile tilapia) is considered an important cheap protein source in Egypt. Heavy clinostomiasis infections result in retarded growth, aberrant behavior, and mortality in fish hosts [3]. *Clinostomum complanatum* and *C. phalacrocoracis* are fish-borne zoonotic parasites known to cause Halzoun syndrome, a rare condition transmitted through the consumption of raw or undercooked freshwater fish infected with *Clinostomum* MCs [4].

Fishermen frequently consider fish with accumulated *Clinostomum* MCs parasites unsuitable for human consumption [5]. The presence of *Clinostomum* on the external skin tissues of the host fish can be easily detected by consumers, often leading to the rejection of fish [6]. However, diagnosing different *Clinostomum* species morphologically is challenging due to their high similarity to each other, with only minor differences in their small and soft-bodied structures [7].

Moreover, a multitude of transcription factors engage with evolutionarily conserved cis-acting regulatory promoter regions to meticulously govern the transcription of both MHC-I and MHC-II genes, a response closely aligned with their pivotal roles. The effective transcription of these genes, tailored to meet the specific demands of a robust immune response, hinges not only on the actions of these regulatory factors but also on the modification of chromatin [8].

In response to parasitic infections, the immune system of the host heightens the production of reactive oxygen species (ROS), encompassing nitric oxide, the superoxide radical (O2-), hydrogen peroxide (H2O2), and the hydroxyl radical (•OH), which function as a line of defense in the resistance of the host against such pathogens. Nevertheless, it is crucial to acknowledge that the reactivity of these ROS with host tissues precipitates oxidative stress [9]. In a recent investigation, a meticulous exploration into the immune status of Nile tilapia naturally afflicted by *Clinostomum* species in Egypt was carried out [1]. Nevertheless, it is crucial to emphasize the existing deficiency in comprehensive investigations concerning the morphological ultrastructure and immunological facets Concerning the various *Clinostomum* spp. found in Egyptian freshwater fish.

This study addresses significant knowledge gaps through several primary objectives. It aims to elucidate the micro-morphological characteristics of *Clinostomum* spp. using advanced scanning electron microscopy, with a primary focus on distinguishing between *C. complanatum* and *C. phalacrocoracis* at the microscopic level. In addition, investigates oxidative stress factors in infected hosts, particularly the production of reactive oxygen species (ROS) like nitric oxide. Furthermore, it explores the complex immune responses triggered by *Clinostomum* infections by examining the host’s immune cytokines. Ultimately, the study seeks to provide valuable insights into the histopathological changes in host tissues and organs resulting from *Clinostomum* infections, contributing to our understanding of the pathophysiological effects caused by these parasites.

## Materials and methods

### Sample collection

A total of four hundred (400) specimens of Nile tilapia (*Oreochromis niloticus*) were gathered from the freshwater environment of the Nile River at Al Bahr Al Aazam, Giza Governorate, Egypt. The collection took place over the course of one year. The collected fish underwent a gross examination to assess the presence of parasitic infections. Fish were transported alive to the parasitology lab using aerated plastic buckets to the laboratories of the Faculty of Veterinary Medicine at Cairo University for comprehensive parasitological and pathological investigations. The examination of the fish encompassed a careful assessment of both external and internal aspects with a focus on identifying *Clinostomum* MCs, parasitic infections, and any associated lesions [10, 11].

### Examination of fish parasites

Each fish specimen underwent a macroscopic examination that included a thorough inspection of the skin, gills, muscle, viscera, and internal organs to detect *Clinostomum* EMCs. The EMCs were manually collected, and according to their dimensions of cysts then assigned two types; small or large-sized cysts were meticulously documented, followed by direct photography. Selected EMCs specimens were excysted using a sterile needle and gently compressed between glass slides. Subsequently, they were fixed in AFA (alcohol–formol–acetic acid) overnight [12]. These specimens were then prepared, stained with Semichon’s acetocarmine, dehydrated through a graded ethanol series, clarified in clove oil, and finally mounted in Canada balsam as permanent preparations [13]. Ten specimens for each type of stained *Clinostomum* species where measurements of the stained specimens were taken followed the criteria [1, 14]. A microscopic examination of the stained specimens was conducted using an Olympus microscope (Japan) to facilitate measurement and description. Photographs of the infected materials and specimens were captured with a Sony digital camera (Japan). The identification of two types of *Clinostomum* species was carried out following the *C. complanatum* and *C. phalacrocoracis* for criteria of identification [11]. Additionally, other whole *Clinostomum* specimens of each species were meticulously preserved at deep freeze (-80 °C) for subsequent molecular analysis.

### Density and distribution of EMCs

This investigation involved estimating the density of EMC in the studied specimens. Furthermore, the distribution of EMC within various anatomical regions of the fish, including the skin, gills, and buccal cavity, was meticulously examined and analysed following the criteria of [15].

### Scanning electron microscopy (SEM)

In the SEM analysis, five specimens from each type of excysted *Clinostomum* species that were prepared as criteria in the established protocol, involved successive rinsing in saline solution and fixation in 2.5% glutaraldehyde, as previously outlined by [16]. Subsequently, the specimens underwent dehydration using an ascending ethanol series, were subjected to drying in a CO_2_ critical point drier (Autosamdri-815, Germany), mounted onto stubs, and finally coated with a 20 nm layer of gold using a sputter coater (Spi-Module Sputter Coater, UK). The prepared specimens were then examined and captured using a scanning electron microscope (SEM) at magnifications ranging from 35X to 500X (JSM 5200, Electron Probe Microanalyzer, JEOL, Japan) at the Faculty of Agriculture, Cairo University.

### Assessment of oxidative stress markers

To evaluate oxidative damage, 10% of the infected tissues from each fish group were homogenized in cold phosphate-buffered saline (pH 7.4) using a homogenizer. The resulting homogenates were then centrifuged at 4000 rpm for 15 min, and the supernatants were stored at -80 °C until further use. The total protein content in the tissue homogenate was determined following the method of [17]. The concentration of Malondialdehyde (MDA) was determined following the method described by [18]. Nitric oxide concentration (NO) was assessed using the procedure outlined by [19].

### MHC-I, MHC-II, and Interleukin-1β analysis

The infected tissues were carefully dissected under stringent hygienic conditions. Conversely, samples from five uninfected, visibly healthy control fish were collected using the same procedures and served as negative controls, following the methods described in previous studies [1, 20]. The infected tissues were used for mRNA isolation, which was performed using total RNA kits from Ambion, Applied Biosystems. Tissue homogenization was carried out with a FastPrep-24 homogenizer (MP Biomedicals) involving two cycles of 30 s at 6 m/s. The purity and quality of the isolated mRNA were evaluated using Nanodrop (Thermo Scientific). Subsequently, 500 nanograms of RNA underwent further processing, including treatment with DNaseI amplification grade from Invitrogen. The High-Capacity cDNA Archive Kit from Applied Biosystems was employed for the reverse transcription of the treated RNA, following the protocols outlined in prior studies [21, 22].

### The quantitative real-time PCR protocol (qRT-PCR)

Primers specifically designed for MHC-I, MHC-II, and Interleukin-1β were used. The primer selection for fish immunity genes was based on sequences available in GenBank (Table 1). To normalize the samples, β-actin was utilized as a reference gene. Each primer was tested on a distinct pool of cDNA, with five samples tested for each primer. Additionally, five non-infected fish, previously examined for the presence of any parasites, were employed as negative controls. The qRT-PCR protocol was carried out according to established procedures [1].

**Table 1:** Primers used for gene expression in tissues of *Clinostomum*-infected fish

Genes	Primers sequences Forward (5'–3')	Primers sequences Reverse (5'–3')	Reference
***IL-1β***	AACACTGACAGAACAACTGCGAACA	TCGCAGTTGTTCTGTCAGTGTTGTT	[20]
***MHC-Ia***	TTCTCACCAACAATGACGGG	AGGGATGATCAGGGAGAAGG	
***MHC-II***	AGTGTGGGGAAGTTTGTTGGAT	ATGGTGACTGGAGAGAGGCG	
***β-Actin***	GAGCGTGAGATTGTGCGTGAC	TCCATACCGAGGAATGAGGGC	

### Statistical analysis

The data obtained from the analysis of oxidative stress and gene expression were subjected to statistical analysis using Predictive Analytics Software (PASW) Statistics and SPSS Version 28 (SPSS Inc., Chicago, IL, USA) [1]. A significance level of *p* < 0.05 was considered to indicate statistical significance. The Chi-Square test was employed to assess potential significant associations between variables.

### Histopathological examination

Metacercariae of *Clinostomum* specimens and the adjacent tissues were meticulously collected from the skin, gills, and buccal cavities of infected fish. These specimens were then fixed in 10% buffered formalin and processed following the procedures outlined by [23]. Subsequently, the sections were deparaffinized and subjected to hematoxylin and eosin (H&E) staining for histological analysis under a light microscope. The muscles and affected organs were examined and documented using an Olympus CX41 microscope.

## Results

### Field and laboratory studies

**Fig. 1:**
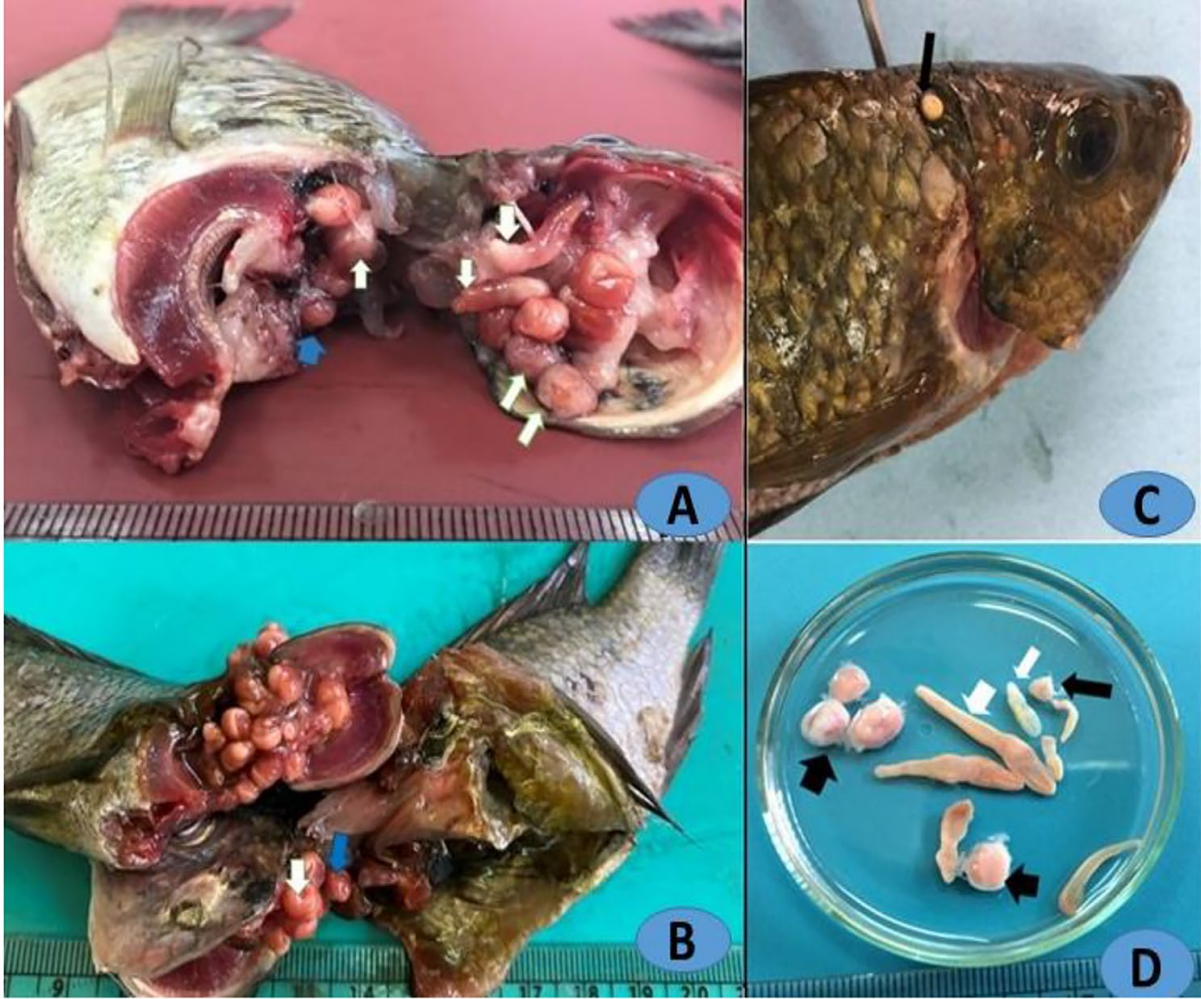
(**A**-**B**): Buccal cavity of investigated *O. niloticus* infected with *Clinostomum* sp. (**A**-**B**) Co-infections with *C. phalacrocoracis*; EMCs of and ExMC (white arrows), EMCs of C. *complanatum* (blue arrow). (**C**) The skin of *O. niloticus* infected with cyst shows *Clinostomum* EMC (arrow). (**D**) *Clinostomum* MCs; *C. complanatum* cyst (thin white arrow), metacercaria (thick white arrows), *C. phalacrocoracis* MC (thick white arrow), and cysts of *C. phalacrocoracis* (thick arrow)

Among the 400 *O. niloticus* specimens examined, 160 individuals (40.0%) were found to harboured larval *Clinostomum* infections. The *Clinostomum* spp. were observed in various anatomical locations, with 135 fish (33.8%) occurring within the buccal cavities, 21 fish (5.25%) in the gill chambers, and 4 fish (1.0%) on the skin. Infection intensity ranged from 2 to 12 cysts per fish with a mean intensity of 5 cysts per fish. Skin infections were notable for mostly having only one cyst per fish in each infected fish (Fig. 1).

### Parasitological examination

*Clinostomum* EMCs were easily discernible to the naked eye, adhering to the skin or located in the buccal cavities upon dissection (Fig. 1A-B). The *Clinostomum* MCs displayed a range of colors, shifting from pale yellowish to yellow. Morphological identification confirmed that they belonged to the species *C. complanatum* and *C. phalacrocoracis*. Notably, externally attached *Clinostomum* EMCs on the host’s skin tissue exhibited a yellowish appearance (Fig. 1C). The highest prevalence of cysts was observed in the buccal cavities, followed by the gill chambers. Within the buccal cavity, two distinct sizes of cysts were noted, with larger rounded cysts (Fig. 1A) and smaller elliptical cysts (Fig. 1B). *Clinostomum* sp. in both excysted (Ex) and encysted (E) forms were also identified, encompassing two size categories: long and short ExMCs (Fig. 1D).

#### Clinostomum metacercariae

The primary morphological features of ExMCs for both *C. phalacrocoracis* and *C. complanatum* were characterized by their stout, tongue-shaped bodies, flat and non-segmented structures with smooth lateral margins, and complete absence of spines. The oral sucker was located terminally, while the ventral sucker occupied the anterior third of the worm’s body. The tandem arrangement of testes was positioned between the midsection and the posterior third of the body. The shape and location of the testes, as well as the poached cirrus, and the characteristics of the ovary’s shape and placement, were identified as the primary features essential for the species differentiation of *Clinostomum* sp.

### Light and scanning electron Microscopy (SEM)

#### Clinostomum phalacrocoracis

**Fig. 2:**
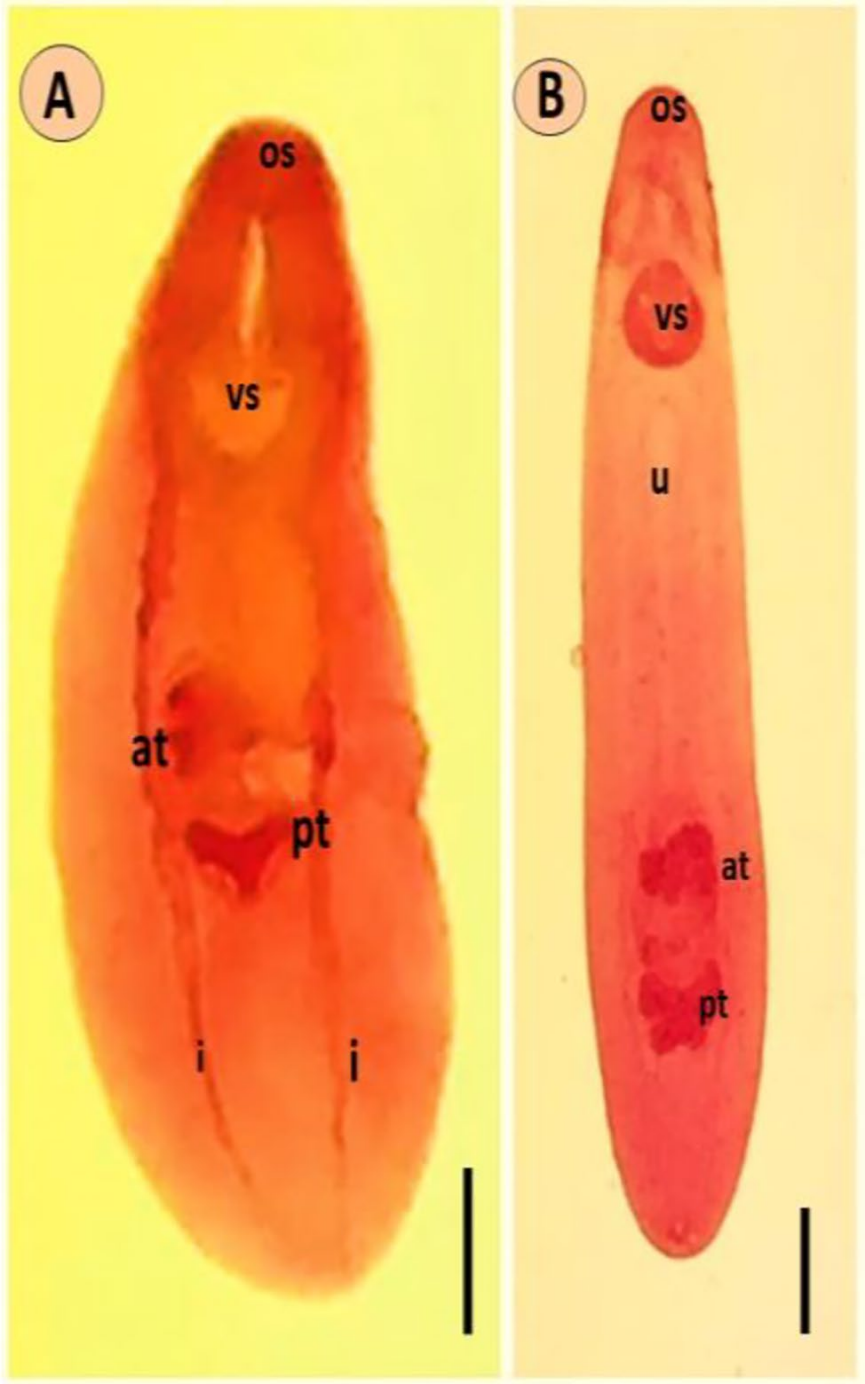
*Clinostomum* species. (**A**) Stained *C. complanatum* metacercaria displaying key anatomical features. (**B**) *C. phalacrocoracis* metacercaria with labelled oral sucker (os), ventral sucker (vs.), uterine tube (u), intestinal ceca (i), anterior testis (at), and posterior testis (pt). Scale bars: (**A**) 2 mm, (**B**) 1.5 mm

**Fig. 3:**
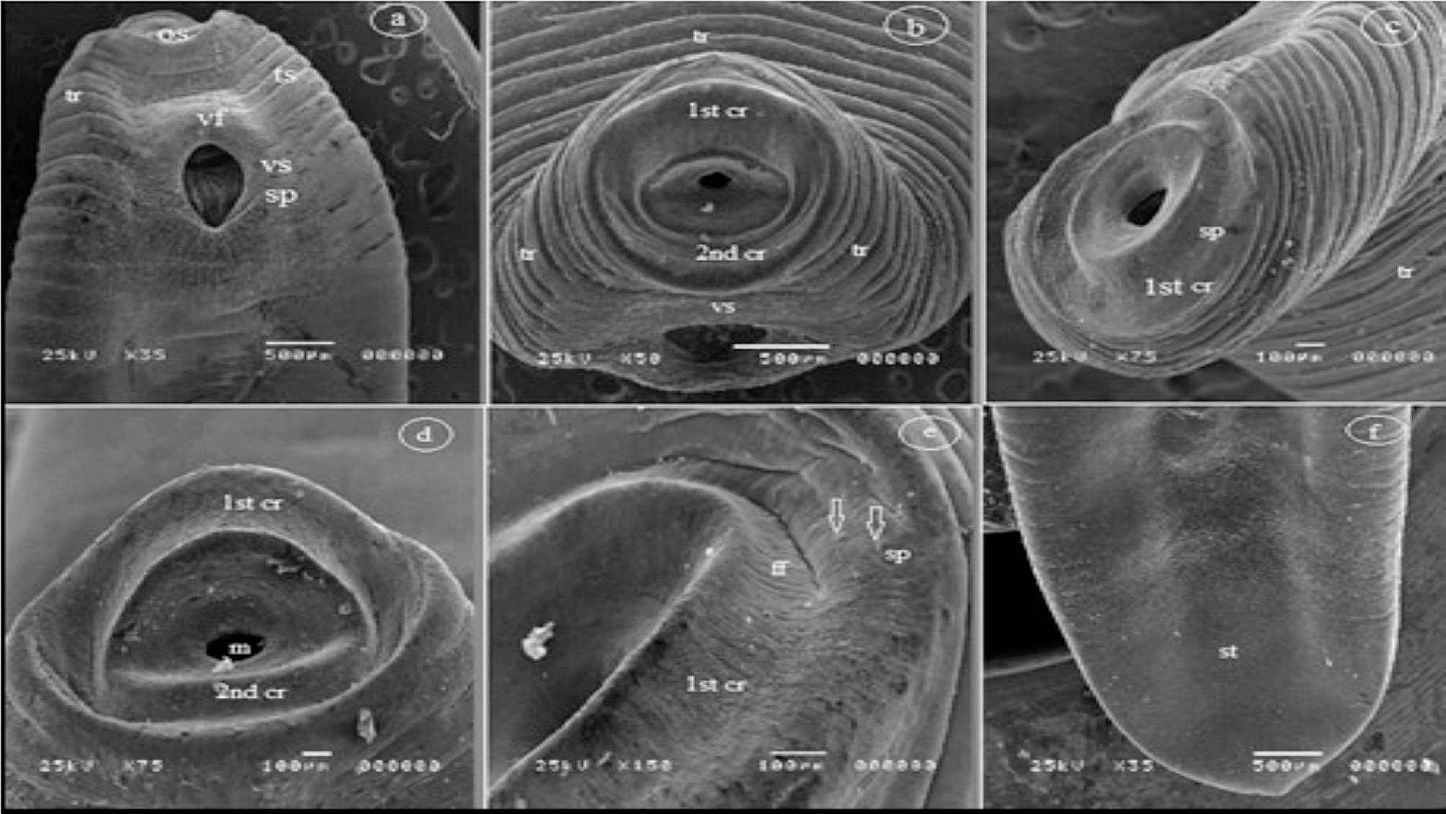
Scanning Electron Micrograph of a *C. phalacrocoracis* ExMC. (**a**) Ventral view of the forebody with the oral sucker (os) and ventral sucker (vs.) indicated. (**b**-**c**) Apical views display distinct dorsal and lateral transverse annulations and ridges (tr) and a terminal circular oral sucker (os). (**d**) Terminal oral sucker with two collar-like rings (1st cr & 2nd cr). (**e**) High-magnification image of the oral sucker showing distinct oral furrows (ff) and sensory papillae (p) (arrows). (**f**) Dorsal tegument of the hind body featuring a smooth tegument (st)

Both excysted metacercariae (ExMC) and encysted metacercariae (EMCs) (Fig. 2A), based on the examination of 10 specimens, exhibited dimensions ranging from minimum to maximum. The larger EMC measured 4.2–8.1 mm in diameter. The ExMC featured a stout body, slightly wider in the gonadic region. Following the oral sucker, there was a short prepharynx that expanded to form a bulb. The pharynx was visibly present, and the intestine bifurcated directly posterior to the pharynx. The intestinal caeca extended laterally to the ventral sucker and reached the posterior extremity of the worm’s body. The testes were branched and positioned in tandem, occupying the posterior third of the body. The anterior testis exhibited a fan-shaped structure and was located between the middle and posterior third of the body, consisting of six to eight blunt lobes. The posterior testis also displayed a fan-shaped configuration with a concave anterior margin and featured two major lateral lobes and one posterior lobe, each of which was subdivided. The genital opening had observable small blunt tubercles along its internal edge (Table 2; Fig. 2A). Ultrastructural examination of the tegumental surface of *C. phalacrocoracis* worms through SEM revealed that the forebody’s tegument displayed distinct regular transverse annulations and ridges, particularly on the dorsal surface (Fig. 3A, B, C). The ventral tegument of the forebody (Fig. 3A) housed a large ventral sucker situated near the oral sucker, with evident transverse annulations and ridges on the lateral sides of the worms. Both the dorsal and ventral lateral sides were devoid of spines. The oral sucker had two distinct collar-like rings; the first collar-like ring featured the typical hemisphere shape, while the second collar-like ring was flat (Fig. 3D). These collar-like rings were equipped with sensory papillae and furrows (Fig. 3E). The ventral sucker was encircled by a ventral fold with a sponge-like texture, and dome-like papillae were present around the margins of the fold (Fig. 3A). In contrast, the hind body displayed a smooth and unaltered tegumental structure (Fig. 3F).

**Table 2:** Measurements of the collected encysted metacercaria

Body parameters	C. complanatum Min–max (mean) (mm)	C. phalacrocoracis Min–max (mean) (mm)
Body length	12.0–14.0 (12)	17.11-23.00 (19.92)
Body width	3.0–4.0 (3.8)	2.82–5.2 (3.2)
Oral sucker (OS) length	0.11–0.31 (0.21)	1.0 -1.3 (1.1)
OS width	0.16–0.33 (0.23)	1.0-1.4 (1.3)
Ventral sucker (VS) length	0.27–0.49 (0.41)	1.1–1.9 (1.2)
VS width	0.46–0.56 (0.43)	1.16–2.1 (1.31)
Anterior testis (AT) length	0.23–0.35 (0.29)	1.6– 2.1 (2.0)
AT width	0.42–0.52 (0.47)	1.9–2.2 (2.1)
Posterior testis (PT) length	0.05–0.18 (0.1)	1.9–2.3 (2.0)
PT width	0.12–0.15 (0.13).	1.9–2.4 (2.2)
Ovary (OV) length	0.04–0.07(0.05)	0.22–-0.35 (0.23)
OV width	0.01–0.03 (0.02)	0.19–0.36 (0.22)

#### Clinostomum complanatum

**Fig. 4:**
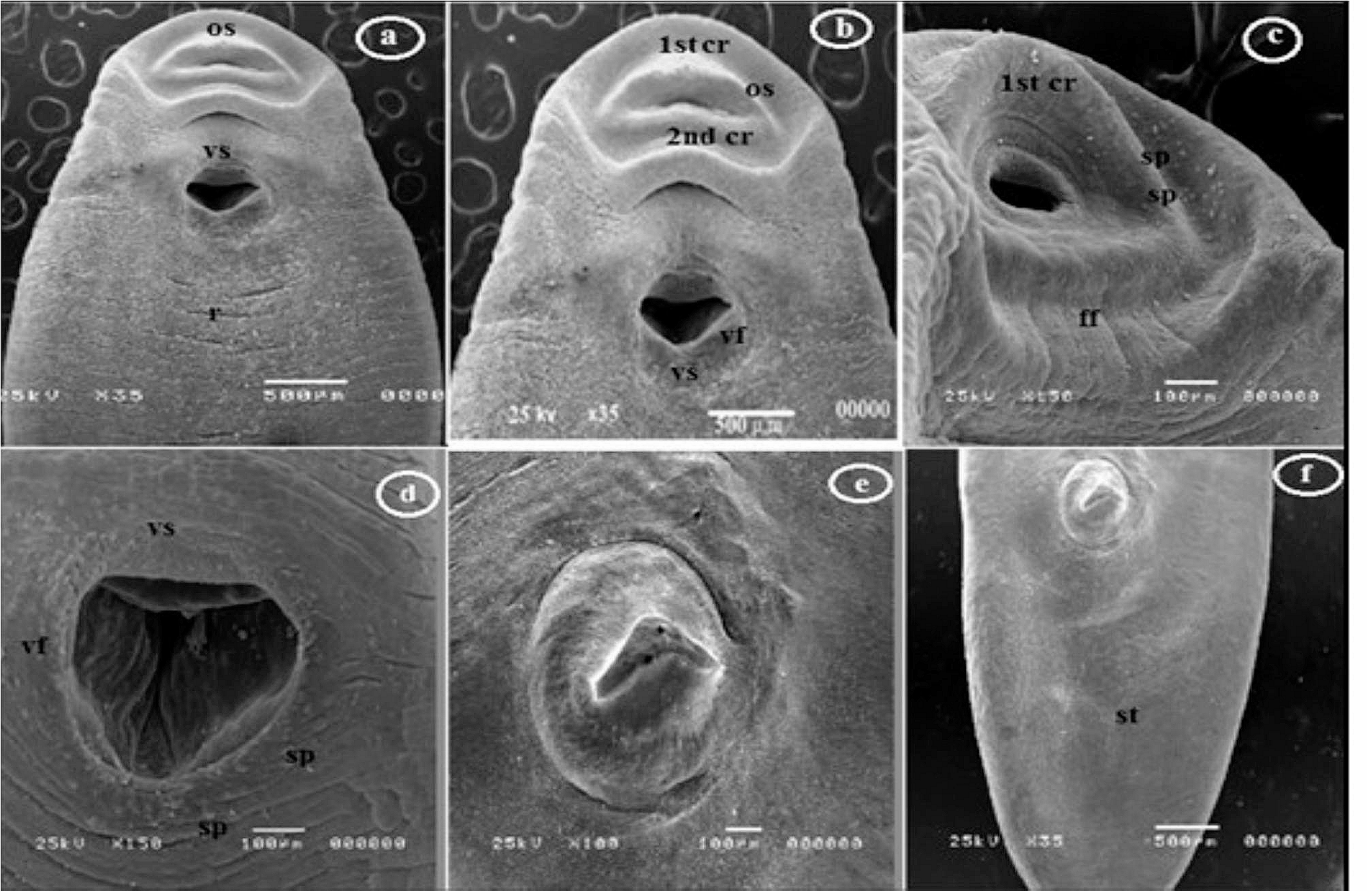
Scanning Electron Micrograph of a *C. complanatum* ExMC. (**a**-**b**) Ventral view of the forebody, revealing an elliptical oral sucker (os), and two collar-like rings (1st cr & 2nd cr). (**c**) Close-up view showing oral furrows (ff) and sensory papillae (sp). (**d**) Ventral sucker (vs.) featuring a spongy ventral fold (vf) and distinct sensory papillae (sp). (**e**) Large sensory papilla-like structures resembling styloconic sensilla in the anterior third of the body. (**f**) Hind body displaying a smooth tegument (st)

The small encysted metacercariae of *Clinostomum* displayed dimensions ranging from 1.0 to 3.0 mm. In contrast, the EMCs were relatively longer, measuring from 11.0 to 14.8 mm (mean: 11.7 mm) in length and 1.8 to 2.2 mm (mean: 1.9 mm) in width (Fig. 2B). These *Clinostomum* MCs exhibited worm-like bodies that resembled a tongue shape and featured bluntly rounded anterior and posterior ends. The testes within the genital complex were located in the middle third of the body, and the posterior testis exhibited a y-like shape, measuring from 0.05 to 0.18 mm (mean: 0.1 mm) in length and 0.12 to 0.15 mm (mean: 0.13 mm) in width. A small ovary was positioned between the testes on the right side, located between the cirrus and uterus (Table 2; Fig. 2B). Upon the ultrastructural examination of the tegumental surface using scanning electron microscopy (SEM), the forebody of the worm exhibited less distinct transverse annulations and ridges, particularly on the ventral and lateral sides of the metacercaria (Fig. 4A). The ventral sucker was positioned near the oral sucker, and the tegumental surface did not feature spines. The oral sucker had an elliptical shape and comprised two distinct collar-like rings. The first collar-like ring was elliptical, while the second collar-like ring had a W-like shape (Fig. 4A-B). These collar-like rings were equipped with sensory papillae and furrows (Fig. 4C). The ventral sucker was encircled by a ventral fold with a sponge-like texture, and dome-like papillae were observed around the margins of the fold (Fig. 4D). An unusual sensory papilla-like structure, resembling styloconic sensilla, was located at the anterior third of the body (Fig. 4E). The hind body displayed a smooth tegumental structure, culminating in a narrow posterior terminal tip (Fig. 4F), which appeared to feature a round excretory hole surrounded by folds.

### Oxidative stress markers

**Fig. 5:**
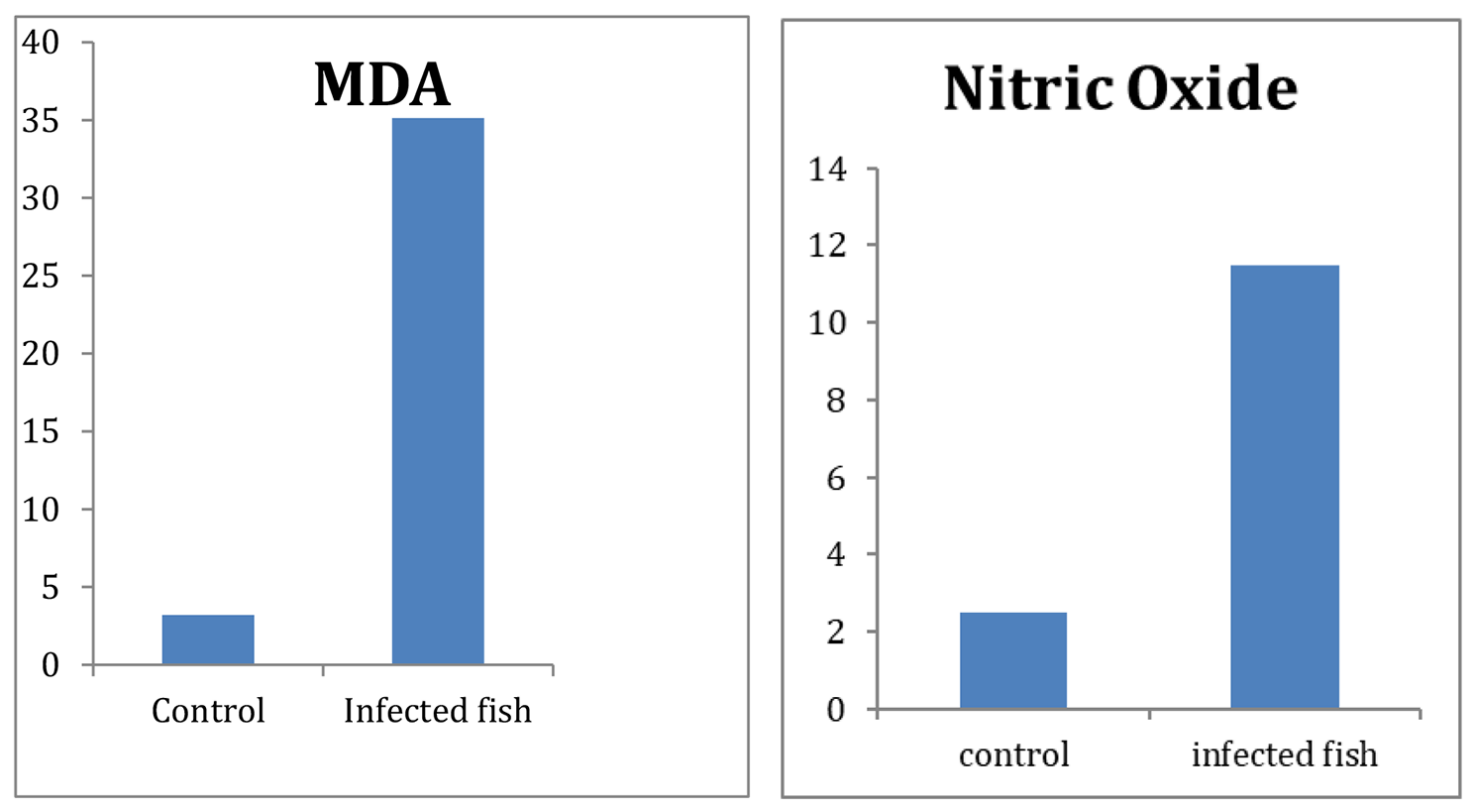
The levels of oxidative stress (MDA) and nitric oxide markers in infected fish with *Clinostomum* sp.

The concentration of malondialdehyde (MDA) in infected fish was markedly elevated compared to that in the non-infected control group. Specifically, the MDA level reached 35.13 ± 6 nmol/g, signifying a substantial increase in oxidative stress in the infected fish. Moreover, the levels of stress markers were notably higher in the infected fish, exemplified by the nitric oxide concentration, which measured 25.80 ± 3.12 µmol/g, in contrast to the negative control, as depicted in (Fig. 5).

### Immune-regulating cytokines

**Fig. 6:**
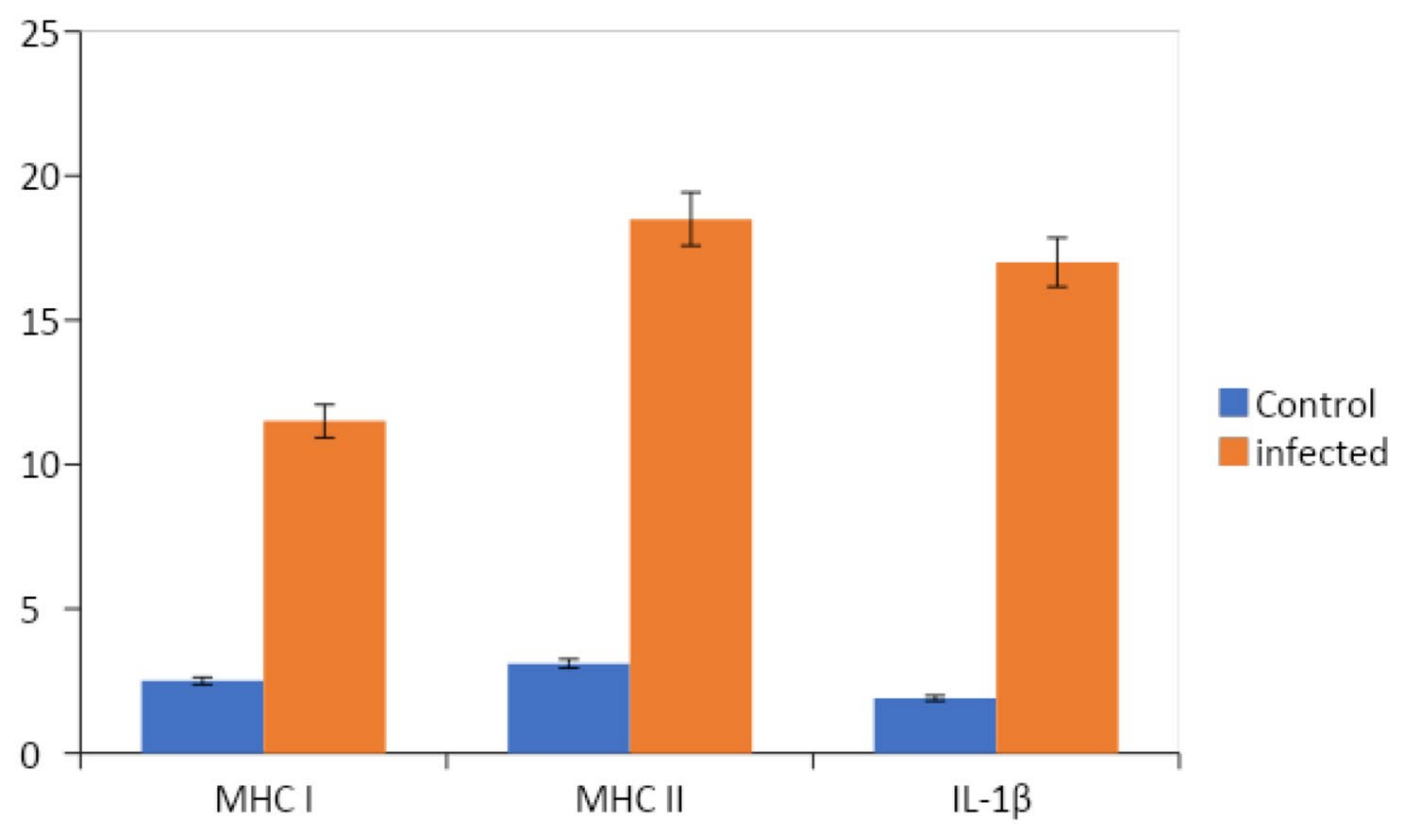
Expression levels of gene markers in *Clinostomum*-infected Fish

In the infected fish, there was a remarkable upregulation in MHC-I gene expression, with mRNA levels exhibiting an approximately 13-fold increase, specifically reaching 11.5 ± 1.2 times higher than those observed in the control group. Moreover, MHC-II gene expression demonstrated a substantial increase in mRNA levels, peaking at 19-fold (with a mean of 18.5 ± 2.3), in contrast to the control group which only exhibited a four-fold increase. Furthermore, the gene expression of IL-1β mRNA showed a significant upregulation, with levels increasing by 17-fold, averaging at 16 ± 3.2, in comparison to the control group where the increase was only two-fold, as depicted in (Fig. 6).

### Histopathological findings

**Fig. 7:**
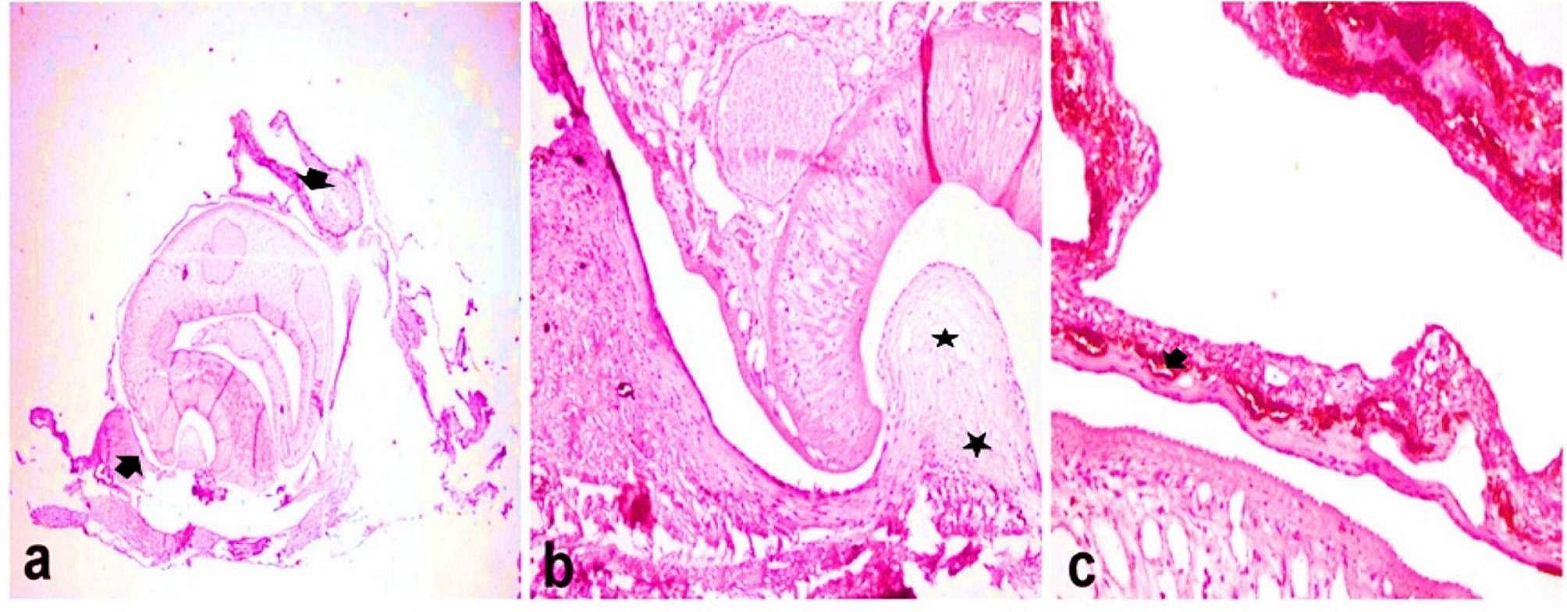
Paraffin sections stained by haematoxylin and eosin (H&E) for histopathological examination of fish skin. (**a**) Large metacercarial cysts firmly attached to cutaneous structures (H&E, 40X magnification, arrows). (**b**) Metacercaria surrounded by a dense layer of connective tissue closely integrated into the host’s cutaneous connective tissue, with focal dispersion and host connective tissue edema (H&E, 200X magnification). (**c**) Activation of melanomacrophages in dermal tissue surrounding the cyst wall (H&E, 400X magnification)

**Fig. 8:**
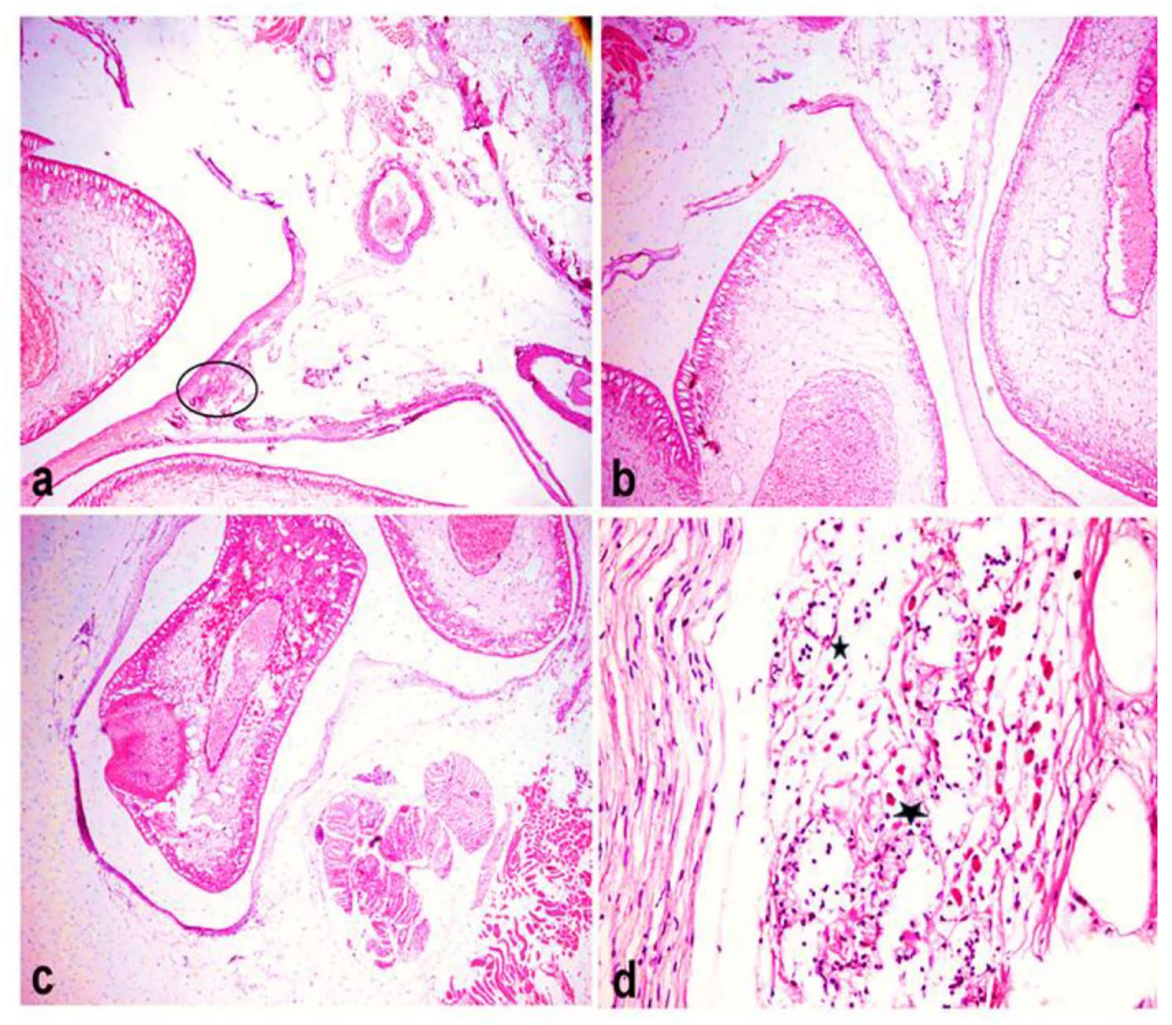
Paraffin sections stained by haematoxylin and eosin (H&E) for histopathological examination of the operculum and pseudobranch of Nile tilapia (**a** & **b**) Presence of two large *Clinostomum* metacercarial cysts enclosed by several layers of collagen firmly attached to the host’s adipose tissue. Note the inflammatory reaction involving the host tissue adjacent to the cyst (circle) (H&E, 40X magnification). (**c**) A metacercarial cyst wall present in the pseudo branch region (H&E, 40X magnification). (**d**) The inflammatory reaction in the host tissue between the two cyst walls consists mainly of EGCs (*) (H&E, X400 magnification)

Microscopic examination of the fish skin showed the presence of cysts containing parasitic metacercaria of *Clinostomum* spp., firmly adhered to subcutaneous structures (Fig. 7a). The cyst wall comprised dense layers of integrated connective tissue, primarily derived from the host’s dermal fibrous tissue (Fig. 7b). Furthermore, the dermal tissue exhibited an accumulation of dermal melanomacrophages (Fig. 7c). Upon microscopic examination of the operculum and pseudo-branch, two cysts containing *Clinostomum* metacercaria were found, firmly anchored to the host tissue through dense collagenous layers (Fig. 8a). These parasitic cysts, housing metacercariae, were encircled by inflammatory reactions, which extended to involve the adipose tissue of fish (Fig. 8b). Within the adipose tissue, notable findings included necrosis, marked aggregation of eosinophilic granular cells (EGCs), extensive necrosis of muscle bundles, and perivascular oedema with fragmentation and vacuolation of the vascular medial wall (Fig. 8c). On the skin of the operculum, hyperplasia of mucous cells and glands that lined the epidermal epithelium was observed. Simultaneously, the underlying dermis exhibited oedema with sparse infiltrating inflammatory cells, predominantly EGCs (Fig. 8d).

## Discussion

Yellow grub disease, caused by *C. complanatum* and *C. phalacrocoracis* metacercariae, represents a widely distributed zoonotic infection in freshwater fishes. While definitive identification of digenean species typically relies on adult fluke morphology, challenges arise in larval stages due to less developed morphological traits and genitalia. To address these challenges, molecular approaches have become crucial for distinguishing morphologically similar or early developmental digenean species [21, 24]. This study employs scanning electron microscopic ultrastructural characterization to delineate *C. phalacrocoracis* and *C. complanatum* MCs infecting *O. niloticus* in Egypt. The integration of morphological and molecular techniques enables precise species-level identification throughout parasite life stages. This comprehensive approach contributes to the ongoing advancement of clinostomid taxonomy, development, and epidemiology [1].

During the parasitological examination in this study, the prevalence of EMCs on the skin of examined fishes was determined to be 1.0%. In contrast, the prevalence of EMCs in the gill chambers and buccal cavities was notably higher, reaching 39.0%. These findings closely align with a prior study on *Clinostomum* spp., reporting a prevalence of 47.5% in buccal cavities and 1.5% on the skin of *O. niloticus* [25]. However, they differ significantly from observations by [26], who documented a considerably higher prevalence of Clinostomum MCs in *O. niloticus* in Egypt, reaching 87.06%. Such variations in prevalence may be attributed to environmental factors, food availability, the abundance of aquatic snails (acting as intermediate hosts), and the presence of aquatic piscivorous birds, which play a pivotal role in the life cycles of certain digenetic trematodes [25, 26].

Within the domain of clinostomid taxonomy, the morphological similarities among various species have posed significant challenges, compounded by the shortage of descriptions of valid species [27]. In this present investigation, the primary morphological characteristics of *C. phalacrocoracis* and *C. complanatum* MCs were examined under light microscopy, focusing on the shape and localization of the testes and the configuration of the cirrus sac as key distinguishing criteria. Notably, the testes of *C. complanatum* assumed a y-shaped pattern, occupying the midsection of the worm’s body, while those of *C. phalacrocoracis* displayed a fan-like shape and were located in the posterior third of the body. These observations resonate with the appearances reported by [28] for *C. complanatum* and [1] for *C. phalacrocoracis*. The fan-shaped anterior testis observed in this study diverges from earlier findings by [16], who characterized the anterior testis as saddle-shaped. This discrepancy may be attributed to differences in the maturity of the metacercariae, as suggested by [29], who noted reduced digitations in the testes of younger specimens. Furthermore, among the various morphometric parameters, including length, the *C. phalacrocoracis* specimens we collected were larger than those documented by [1]. This variability in metacercarial size may arise from differences in the age of the parasites acquired or could be influenced by the contraction of the body of the specimen. The location of the cirrus sac in *C. complanatum* MCs, as identified in this study, was situated to the right of the anterior testis, consistent with the descriptions provided by [30]. It is noteworthy that the shape of the cirrus sac can undergo alterations during development [31]. Furthermore, distinctions between species can be drawn based on the measurements of the distance between the oral and ventral suckers and body width. However, these structural dimensions may exhibit variability depending on the developmental stage of the MCs, host-specific factors, and the procedures involved in specimen preparation for microscopic examination [15, 16].

In this study, distinctions between *C. complanatum* and *C. phalacrocoracis* were made through the first comparative ultrastructural examination of tegument topology using scanning electron microscopy (SEM). The tegumental surface of both *Clinostomum* species exhibited similar key features, including a smooth texture devoid of spines, sensory papillae, furrows, and spongy underlying tissue. These distinctions align with previous morphological descriptions of *Clinostomum* lacking body spines from SEM-based studies conducted in Egypt, Indonesia, and Thailand [32, 33]. The smooth tegumental surface observed contrasts with an earlier light microscopy report by [34] in Egypt, noting minute spines covering *Clinostomum* bodies, except for the posterior fourth. These distinctions, based on electron microscopic evidence, indicate that spineless teguments are characteristic of *Clinostomum* species across different geographical regions. Further comparative ultrastructural studies on *Clinostomum* from diverse hosts and locations are warranted to confirm uniformity across the genus and elucidate fine-scale topographical distinctions between species.

The current investigation revealed that the tegumental surface of *Clinostomum* MCs presents a smooth texture with sensory papillae, furrows, and a spongy tissue layer, consistent with previous SEM-based descriptions, particularly by [33]. Additionally, a distinctive characteristic was observed in the oral sucker, with *C. phalacrocoracis* displaying a circular shape and *C. complanatum* exhibiting an elliptical form. Sensory papillae and furrows were evident at the base of the tegumental surface in both species, in agreement with the findings of [35] in Indonesia, who noted an elliptical oral sucker in *C. complanatum* and reported the presence of sensory papillae and furrows. It’s worth noting that [32] observed wrinkles on the oral sucker of *Clinostomum* species, a feature they indicated was absent in other trematodes.

The marked elevation in malondialdehyde (MDA) concentration to 35.13 ± 6 nmol/g in Nile tilapia infected with *Clinostomum* MCs compared to non-infected controls indicates substantial oxidative stress induced by the parasite [36]. MDA is a well-established marker of lipid peroxidation resulting from reactive oxygen species-mediated damage [37]. The significant increase in nitric oxide (NO) levels, reaching 25.80 ± 3.12 µmol/g in infected Nile tilapia compared to controls, highlights the pronounced oxidative stress caused by *Clinostomum* infection. Nitric oxide is a key immune signaling molecule produced in response to tissue damage and inflammation [38]. This rise in nitric oxide reflects an active immune response against the parasite. The parasitic infection triggers nitric oxide production, likely as a defense against the parasite’s damage and to regulate inflammation [39]. This insight into nitric oxide dynamics sheds light on oxidative stress and immune reactions in Clinostomiasis. These results clearly show that *Clinostomum* infection imposes significant oxidative challenges on Nile tilapia hosts. Infestations by parasites can lead to oxidative stress, inflict physical harm, and induce degenerative alterations in the organs of fish. These effects arise from an imbalance between pro-oxidants and enzymatic or non-enzymatic antioxidants [40]. Oxidative stress has detrimental impacts including impaired osmoregulation, reduced growth, muscle degradation, and immunosuppression [36]. The mobilization of antioxidant systems to mitigate these effects may divert energy from other physiological needs [41].

The current study has unveiled the activation of immune-modulatory cytokines, specifically IL-1β, MHC I, and MHC II. This insight provides valuable information on the physiological responses within fish hosts during infection with the two metacercarial species under investigation. The upregulation of these pro-inflammatory processes aims to regulate and mitigate potential harmful consequences resulting from an excessive inflammatory reaction. These processes are closely intertwined with cytokines critical for immune responses, particularly in the context of anti-parasitic immunity, as emphasized by [42]. Notably, significant disparities in the concentrations of cytokines and chemokines were observed between infected and uninfected fish hosts [1].

Histopathological inspection of the skin tissue revealed the presence of *Clinostomum* MC within the subcutaneous layers, coinciding with the absence of the epidermal stratum. The structural composition of the cyst encompassed densely woven layers of fibrous connective tissue, primarily originating from the dermal tissues of the host, securely attached to the underlying cutaneous structures. The considerable thickness of the cyst wall, in terms of histopathological interpretation, serves as an indicator of a relatively advanced stage of infection and lesion maturation. This attribute is well-documented and has been elucidated by [7, 8]. The detrimental effects of *Clinostomum* on the integumentary tissues and ocular structures of fish have been extensively documented in previous studies [43]. Moreover, [44] documented the presence of cysts of *Clinostomum* parasites beneath the skin in the cranial region of catfish.

Histopathological findings from the study revealed the presence of cysts attached to the operculum and pseudo branch of the fish. Existing literature has consistently identified the operculum and oral cavity as primary sites for cercarial attachment, with some authors postulating that the elevated incidence in these regions is influenced by water currents, fostering cercarial breeding activity and increasing the likelihood of contact [7, 8]. A higher occurrence of *C. complanatum* is observed in the region extending from beneath the mouth to behind the operculum [45]. Microscopic analysis during the investigation unveiled that the parasitic cyst harboring metacercariae was concealed within collagen sheets of relatively thin thickness, signifying a recent infestation of the fish by the parasite. It’s noteworthy that the thickness of the cyst wall serves as an indicator of the duration of infection [1].

The histopathological changes induced by the presence of *Clinostomum* metacercariae within fish tissue were characterized by substantial inflammatory reactions that impacted the host structure. These reactions were primarily manifest through the infiltration of eosinophilic granular cells (EGCs), myocytolysis, and the necrosis and degeneration of fat cells [15, 16]. Furthermore, the skin of the operculum exhibited pronounced hyperplasia of mucous cells, reflecting a pathological response within the fat and musculature tissues of fish. This finding aligns with previous observations by [43], who noted a similar detrimental reaction induced by *Clinostomum* parasites in fish. It is noteworthy that fish tend to develop cysts around parasites as a response to infection. This reaction results in irritation and the stimulation of mucous secretions in the skin of fish surrounding the cyst wall [46].

## Conclusion

This study presented a novel perspective on Clinostomum infections in Nile tilapia using an integrated approach that combined ultrastructural description, oxidative stress markers, immunology, and histopathology. Scanning electron microscopy enabled detailed visualization of tegumental features like sensory papillae and subsurface spongy tissue, differentiating between *C. complanatum* and *C. phalacrocoracis*. Elevated malondialdehyde and nitric oxide indicated oxidative damage and inflammation induced by *Clinostomum*. Upregulated MHC-I, MHC-II, and IL-1β showed host immune responses, though ineffective in parasite clearance. Histopathological lesions in skin and buccal cavity tissues aligned with the destructive inflammatory response and cyst encapsulation from infection. These initial insights into the parasite-host interaction in clinostomiasis set the stage for further research. A comprehensive investigation using advanced morphological, molecular, cellular, and immunological techniques was essential to advance *Clinostomum* taxonomy, elucidate host-parasite dynamics, and develop sustainable disease control strategies. This research was crucial to mitigate risks from this emerging zoonosis impacting aquaculture, food security, and human health.

## Data Availability

All data generated or analysed during this study are included in this paper.
